# Intranasal influenza-vectored COVID-19 vaccines confer broad protection against SARS-CoV-2 XBB variants in hamsters

**DOI:** 10.1093/pnasnexus/pgae183

**Published:** 2024-05-03

**Authors:** Junyu Chen, Congjie Chen, Lunzhi Yuan, Yaode Chen, Xijing Wang, Ningxin Tang, Dongmei Wei, Xiangzhong Ye, Ningshao Xia, Yixin Chen

**Affiliations:** State Key Laboratory of Vaccines for Infectious Diseases, Xiang An Biomedicine Laboratory, Department of Laboratory Medicine, School of Public Health, School of Life Sciences, Xiamen University, No.4221, Xiang'an South Road, Xiang'an District, Xiamen 361102, China; National Institute of Diagnostics and Vaccine Development in Infectious Diseases, State Key Laboratory of Molecular Vaccinology and Molecular Diagnostics, Collaborative Innovation Center of Biologic Products, National Innovation Platform for Industry-Education Integration in Vaccine Research, Xiamen University, No.4221, Xiang'an South Road, Xiang'an District, Xiamen 361102, China; State Key Laboratory of Vaccines for Infectious Diseases, Xiang An Biomedicine Laboratory, Department of Laboratory Medicine, School of Public Health, School of Life Sciences, Xiamen University, No.4221, Xiang'an South Road, Xiang'an District, Xiamen 361102, China; State Key Laboratory of Vaccines for Infectious Diseases, Xiang An Biomedicine Laboratory, Department of Laboratory Medicine, School of Public Health, School of Life Sciences, Xiamen University, No.4221, Xiang'an South Road, Xiang'an District, Xiamen 361102, China; National Institute of Diagnostics and Vaccine Development in Infectious Diseases, State Key Laboratory of Molecular Vaccinology and Molecular Diagnostics, Collaborative Innovation Center of Biologic Products, National Innovation Platform for Industry-Education Integration in Vaccine Research, Xiamen University, No.4221, Xiang'an South Road, Xiang'an District, Xiamen 361102, China; State Key Laboratory of Vaccines for Infectious Diseases, Xiang An Biomedicine Laboratory, Department of Laboratory Medicine, School of Public Health, School of Life Sciences, Xiamen University, No.4221, Xiang'an South Road, Xiang'an District, Xiamen 361102, China; State Key Laboratory of Vaccines for Infectious Diseases, Xiang An Biomedicine Laboratory, Department of Laboratory Medicine, School of Public Health, School of Life Sciences, Xiamen University, No.4221, Xiang'an South Road, Xiang'an District, Xiamen 361102, China; State Key Laboratory of Vaccines for Infectious Diseases, Xiang An Biomedicine Laboratory, Department of Laboratory Medicine, School of Public Health, School of Life Sciences, Xiamen University, No.4221, Xiang'an South Road, Xiang'an District, Xiamen 361102, China; State Key Laboratory of Vaccines for Infectious Diseases, Xiang An Biomedicine Laboratory, Department of Laboratory Medicine, School of Public Health, School of Life Sciences, Xiamen University, No.4221, Xiang'an South Road, Xiang'an District, Xiamen 361102, China; Beijing Wantai Biological Pharmacy Enterprise Co., Ltd., No.31, Kexueyuan Road, Changping District, Beijing 102206, China; State Key Laboratory of Vaccines for Infectious Diseases, Xiang An Biomedicine Laboratory, Department of Laboratory Medicine, School of Public Health, School of Life Sciences, Xiamen University, No.4221, Xiang'an South Road, Xiang'an District, Xiamen 361102, China; National Institute of Diagnostics and Vaccine Development in Infectious Diseases, State Key Laboratory of Molecular Vaccinology and Molecular Diagnostics, Collaborative Innovation Center of Biologic Products, National Innovation Platform for Industry-Education Integration in Vaccine Research, Xiamen University, No.4221, Xiang'an South Road, Xiang'an District, Xiamen 361102, China; State Key Laboratory of Vaccines for Infectious Diseases, Xiang An Biomedicine Laboratory, Department of Laboratory Medicine, School of Public Health, School of Life Sciences, Xiamen University, No.4221, Xiang'an South Road, Xiang'an District, Xiamen 361102, China; National Institute of Diagnostics and Vaccine Development in Infectious Diseases, State Key Laboratory of Molecular Vaccinology and Molecular Diagnostics, Collaborative Innovation Center of Biologic Products, National Innovation Platform for Industry-Education Integration in Vaccine Research, Xiamen University, No.4221, Xiang'an South Road, Xiang'an District, Xiamen 361102, China

## Abstract

The XBB.1.5 subvariant has garnered significant attention due to its exceptional immune evasion and transmissibility. Significantly, the evolutionary trajectory of SARS-CoV-2 has shown continual progression, with a recent global shift observed from XBB to BA.2.86, exemplified by the emergence of the predominant JN.1 subvariant. This phenomenon highlights the need for vaccines that can provide broad-spectrum antigenic coverage. In this study, we utilized a NS1-deleted (dNS1) influenza viral vector to engineer an updated live-attenuated vectored vaccine called dNS1-XBB-RBD. This vaccine encodes the receptor-binding domain (RBD) protein of the XBB.1.5 strain. Our findings demonstrate that the dNS1-XBB-RBD vaccine elicits a similar systemic and mucosal immune response compared to its prototypic form, dNS1-RBD. In hamsters, the dNS1-XBB-RBD vaccine provided robust protection against the SARS-CoV-2 immune-evasive strains XBB.1.9.2.1 and Beta. Remarkably, nasal vaccination with dNS1-RBD, which encodes the ancestor RBD gene, also effectively protected hamsters against both the XBB.1.9.2.1 and Beta strains. These results provide valuable insights about nasal influenza-vectored vaccine and present a promising strategy for the development of a broad-spectrum vaccine against COVID-19 in the future.

**Significance Statement**

COVID-19 is still circulating worldwide, and remains a global concern. Relying solely on updated vaccine candidates for prevention against SARS-CoV-2 infection poses challenges due to the incessant emergence of variants. In this study, we report that both the updated XBB.1.5 derived dNS1-XBB-RBD vaccine and the ancestral dNS1-RBD vaccine could establish RBD-specific adaptive and cellular immune response, maintaining the local tissue-resident memory T-cell response, and trained immunity. In hamster challenge model, the dNS1-XBB-RBD vaccine proved be effective to cope with Beta and XBB.1.9.2.1. Importantly, the dNS1-RBD vaccine could also provide effective protection against lung pathology induced by the antigenically distant XBB.1.9.2.1 variant.

## Introduction

Despite the rapid development and clinical deployment of COVID-19 vaccines, the SARS-CoV-2 XBB.1.5 subvariant (BA.2), a descendant of XBB, has emerged as an exceptionally efficient and highly contagious strain (1, 2). This subvariant evades humoral immunity induced by vaccination and natural infection to a significant extent (3, 4). It has now replaced previously dominant omicron variants worldwide and is rapidly becoming the prevailing strain since its first discovery in late 2022. However, the emergence of numerous escape variants indicates that COVID-19 will persist in the human population for years to come (5, 6). During fall 2023, XBB lineages cocirculated with JN.1, an Omicron BA.2.86 lineage with increasing escape from neutralizing antibodies (7, 8). The XBB lineages predominated until December 2023, at which point the JN.1 strain became the dominant variant first in the United States and subsequently globally. The rapid mutation characteristics of SARS-CoV-2 have furthermore led to the emergence of new immune escape variants as the main prevalent strains shortly after the XBB vaccine was licensed (9). Thus, achieving prevention against SARS-CoV-2 infection solely through updated vaccination is challenging due to the continuous emergence of variants and their “anatomical escape” characteristics (10–12), which demonstrated that antibody levels in the respiratory tract are 200–500 times lower than that in circulation system.

Therefore, there is a need to encourage the development of broad-spectrum COVID-19 vaccines that utilize different immune mechanisms and technical approaches. Theoretically, locally induced protective immune responses through nasal or inhaled SARS-CoV-2 vaccines could respond to SARS-CoV-2 infection more rapidly than immune effectors found in peripheral lymph nodes and blood, particularly in the respiratory tract (13–15). Globally, approximately 100 different mucosal vaccines against SARS-CoV-2 are currently in development (16–18). Previously, we developed an intranasal COVID-19 vaccine called dNS1-RBD, based on live-attenuated influenza viral vectors (19–21). This vaccine has demonstrated a broad-spectrum protective effect against symptomatic Omicron infection in a randomized placebo-controlled Phase III clinical trial (22). As a result, it was granted emergency use approval in China on 2022 December 2 (23). This vaccine elicited both specific and nonspecific protective immune responses (19, 20) that covered the upper and lower respiratory tracts. These responses restrained the inflammatory response by reducing early-phase viral load after SARS-CoV-2 infection and attenuating proinflammatory cytokine production. This vaccine represents a promising strategy for achieving broad-spectrum protection and reducing the burden of COVID-19 disease. We know that the bivalent vaccine targeting both the wild-type and BA.4–BA.5 spike proteins of SARS-CoV-2 (Pfizer-BioNTech) has been constructed and licensed (24), following by recommended monovalent XBB.1.5–derived vaccine, for a vaccine efficacy of 54% (95% confidence interval 46–60%) against symptomatic infection (9). As such, the necessity and protective efficacy of an updated dNS1-based nasal vaccine targeting the XBB subvariants remain uncertain.

In this study, we describe the development of an updated NS1-deleted influenza viral vectored vaccine called dNS1-XBB-RBD. This vaccine encodes the RBD protein of the XBB.1.5 strain and can be used for primary or booster immunizations. Additionally, we assessed the systemic, mucosal, and trained immune responses elicited by the dNS1-RBD and dNS1-XBB-RBD vaccines. Furthermore, we evaluated their protective efficacy against challenge with the Beta and XBB.1.9.2.1 variants in Syrian hamster model.

## Materials and methods

### Cell cultures

All cell lines were obtained from American type culture collection (ATCC). Human embryonic kidney cells (293T), African green monkey kidney epithelial cells (Vero E6), and Madin-Darby canine kidney cells (MDCK) were maintained in Dulbecco's modified eagle medium (DMEM)-high glucose (Sigma Aldrich) supplemented with 10% low endotoxin fetal bovine serum (FBS) (Cegrogen Biotech) and penicillin–streptomycin.

### Generation and passage of dNS1-XBB-RBD viruses

The sequence encoding the RBD segment of XBB.1.5 (GenBank accession number WEX63463.1) was codon optimized for eukaryotic expression system and then cloned into the NS1 deletion plasmid pHW2000-DelNS1 as described previously (19). Eight pHW2000 plasmids containing the DelNS1 segment and the other seven influenza virus genomic segments, together with an NS1 expression plasmid, pCX-CA04-NS1-Flag were transfected into 293T cells and incubated overnight at 37°C. The DNA mixture was removed, and Opti-MEM supplemented with 1 μg/mL 6-(1-tosylamido-2-phenyl) ethyl chloromethyl ketone (TPCK)-treated trypsin (Sigma) was added. Viral supernatant was collected 72 h later, designated dNS1-XBB-RBD passage 0 virus, and was subsequently passaged in MDCK cells at 33°C. The supernatant was harvested 48 h post-transfection when most of the cells showed signs of cytopathic effect (CPE). Infectious virus titers (PFU/mL) were determined by plaque assay on MDCK cells.

### Growth kinetics

MDCK cells seeded in 24-well plates were infected with viruses at the indicated multiplicity of infection (MOI). After 1 h of adsorption, the viral supernatant was removed, and the cells were washed twice with phosphate-buffered saline (PBS). DMEM containing 1 μg/mL TPCK-treated trypsin was added, and the cells were incubated at the indicated temperature. Supernatants were collected at different time points, and titers were determined by plaque assay.

### Plaque assay for influenza viruses

Viruses were 10-fold serially diluted, added to confluent MDCK cells in 6-well plates and then incubated at 37°C for 1 h. The supernatant was removed, and the cells were washed twice with PBS and then overlaid with 1% MEM agarose containing 1 μg/mL TPCK-treated trypsin. The plates were incubated at 33°C for 72 h and then fixed with 4% PBS-buffered formaldehyde solution for at least 1 h. Plaques were visualized by staining with 1% crystal violet solution.

### Western blot

MDCK cells were cultured and infected with dNS1-RBD, CA04-WT, dNS1-Vector, and dNS1-XBB-RBD virus as described above. Thirty-six hours later, cell lysates were harvested using modified Nonidet P-40, EDTA and Tris-HCl (NET) cell lysis buffer. Proteins were separated on a 10% gel, and then following transfer, blots were incubated with an anti-influenza A nucleoprotein (NP) protein antibody 19C10 (1:1,000) and anti-RBD antibody R3H2 (1:2,000) generated by our laboratory and visualized with horseradish peroxidase (HRP)-conjugated anti-mouse IgG (Invitrogen, 1:5,000).

### Immunofluorescence imaging

For direct visualization of the expression of hemagglutinin (HA) and RBD, MDCK cells were seeded at 2 × 10^4^ cells per well in CellCarrier-96 Black plates and then infected with dNS1-RBD, dNS1-XBB-RBD, dNS1-Vector, and CA04-WT at an MOI of 1. PBS was used as a negative control. After 72 h, the cells were fixed with 2% paraformaldehyde in PBS for 15 min in the dark. The cells were then permeated by the addition of 0.3% Triton X-100 in PBS (PBST) for 10 min at room temperature and blocked with 2% bovine serum albumin (BSA). The plates were incubated with a DyLight 650-labeled mAb against 6G9-488 (anti-HA; 1:100 dilution) and DyLight 488-labeled mAb against R3H2 (anti-RBD; 1:100 dilution) generated by our laboratory at 37°C for 60 min, and the assay plates were washed three times with PBS. Cell nuclei were labeled with 4′, 6-diamidino-2-phenylindole (DAPI). The images were acquired on an Opera Phenix using a 63× water immersion objective.

### Vaccine formulation

The vaccine dNS1-RBD, dNS1-XBB-RBD, and dNS1-Vector was prepared on large-scale at Beijing Wantai Biological Pharmacy Enterprise Co., Ltd., Beijing, China. After rounds of passage and amplification with the cell factory based on the MDCK cell line, the viruses were further purified through process step by step including ultrafiltration, size-exclusion chromatography, nuclease treatment and then ion-exchange chromatography to confirm the exclusion of exogenous factors. Purified virions were then mixed with virus protectant, which contained carbohydrates, amino acids and human albumin, etc. and were preserved at −15°C. Based on the ELISA results using a sandwich assay with anti-RBD monoclonal antibodies on both sides (Wantai, Beijing, China) and plaque assay results, serial passages 1 to 10 of purified vaccines were confirmed to be stable under current vaccine manufacturing conditions. The purified vaccines were further used in this study for comprehensive evaluation.

### Ethics statements

All animals involved in this study were housed and cared for in an Association for the Assessment and Accreditation of Laboratory Animal Care (AAALAC)-accredited facility. All experimental procedures with mice, rabbits, and hamsters were conducted according to Chinese animal use guidelines and were approved by the Institutional Animal Care and Use Committee (IACUC) of Xiamen University (XMULAC20230240). The hamster studies were performed in an animal biosafety level 3 (ABSL-3) laboratory affiliated to the State Key Laboratory of Emerging Infectious Diseases, The University of Hong Kong.

### Vaccine safety evaluation

The safety of dNS1-XBB-RBD was evaluated in New Zealand rabbits. New Zealand rabbits were intranasally inoculated 200 μL with triple-doses of 10^6^ PFU of dNS1-XBB-RBD or saline under isoflurane anesthesia at 1-week interval and monitored weight changes at days 1, 8, 15, 16, and 29 post-inoculation. Histopathological evaluations for nasal turbinate, trachea, and lungs from the two groups at day 16 postadministration were conducted.

### Immunization and infection of mice

BALB/c and C57BL/6 mice were immunized intranasally with 50 μL of 1 × 10^6^ PFU/mL of the vaccine per dose prepared as indicated above under isoflurane anesthesia. For antibody response evaluation, all groups of BALB/c mice (five animals in each group) were vaccinated by a prime-boost regimen (days 0 and 14), and blood was collected via retro-orbital bleeding 14 days after the second injection, followed by a binding assay to analyze vaccine immunogenicity.

For cellular immune response analyses of pulmonary lymphocytes, C57BL/6 mice (6–8 weeks old) were immunized intranasally with 50 μL of 1 × 10^6^ PFU/mL of the vaccine by two-dose regimen as described above (five animals in each group). Then, pulmonary lymphocytes were collected on day 28 of a prime-boost regimen with a 2-week interval for ELISPOT assay. In tissue-resident T-cell and trained immunity analyses experiment (five animals per group), mice were intranasally immunized with a booster dose on day 14 and pulmonary lymphocytes were harvested on day 14 after boost vaccination. Intracellular cytokine staining (ICS) analyses of pulmonary lymphocytes involved intranasal immunization of C57BL/6 mice with a single dose (52 animals per group), and collection of pulmonary lymphocytes on day 7 after vaccination.

### Flow cytometry

The expression of phenotypic markers, activation markers, and cytokines was evaluated. Briefly, cells were washed and blocked with anti-CD16/CD32 (clone 2.4G2) in 0.5% FBS–PBS for 30 min on ice, then stained with fluorochrome-labeled mAbs for 30 min on ice. ICS assays involved stimulating each sample with pooled spike peptides (1.0 μg/mL) in a U-bottom plate and incubating at 37°C for 18 h. Golgi-Plug (BD Biosciences) was added to the culture at a final concentration of 1:1,000, and cells were further incubated for 6 additional hours. After incubation, cells were washed and stained with fluorochrome-labeled mAbs for 30 min on ice. The stained cells were fixed and permeabilized with BD Cytofix/Cytoperm (BD Biosciences, San Jose, CA, USA) according to the manufacturer's instructions. The cells were washed and intracellularly stained with fluorochrome-labeled mAbs for 45 min on ice. The antibody reagents used in this study include: CD4 [Clone GK1.5, FTIC], CD8a [Clone 53-6.7, PerCP-cy5.5], NK1.1 [Clone PK136, APC/Cyanine7], CD64 [Clone X54-5/7.1, PE/Cy7], CD170 [Clone S17007L, PerCP-cy5.5], CD11b [Clone M1/70, PE], CD86 [Clone PO3, BV605], CD11c [Clone N418, BV421], CD45.2 [Clone 104, BV785], CD103 [Clone2E7, PE], CD69 [CloneH1.2F3, BV421], CD44 [Clone IM7, PE/Cy7], Ly-6C [Clone HK1.4, APC/Cy7], MHC class II [clone M5/114.15.2, APC], cytokine expression (IFN-γ [clone XMG1.2, APC]), and a LIVE/DEAD Fixable Aqua Dead cell stain kit was also used. Stained cells were processed using a BD LSRFORTESSA X-20 (BD Biosciences) flow cytometry system according to the manufacturer's instructions. Data were analyzed using FlowJo X 10.8.1

### Immunization and infection of hamsters

Hamsters (male:female = 1:1, 8 animals in each group) were vaccinated with the indicated amount of the vaccine. All hamsters received 100 μL with 1 × 10^6^ PFU/mL of vaccine per dose via the intranasal route. At the indicated time after vaccination, the hamsters were further evaluated by direct contact challenge of SARS-CoV-2. The B.1.351 variant (AP100, hCoV-19/China/AP100/2021; GISAID accession No. EPI_ISL_2779638) and XBB.1.9.2.1 variant strain (AP-144, share an identical sequence with EPI_ISL_17660518) used in this study were passaged on Vero cells (#CCL-81, ATCC). Virus-carrying hamsters (donors) were preinfected via inoculation of 1 × 10^4^ TCID_50_ of SARS-CoV-2 through the intranasal route. Each donor was transferred to a new cage and cohoused with four vaccinated or control animals. One day after cohousing, donors were isolated from the cage, and the other hamsters were further observed. The hamsters were fed a daily food amount of 7 g per 100 g of body weight. The weight changes and typical symptoms (piloerection, hunched back, and abdominal respiration) in hamsters were recorded daily after virus inoculation or contact. Hamsters were sacrificed for tissue pathological and virological analyses on days 5 and 7 after virus challenge. The virus challenge studies were performed in an ABSL-3 facility.

### Anti-RBD IgA measurements

Bronchoalveolar lavage (BAL) was collected on vaccine-infected mice. Mice were euthanized, and a short needle insulin syringe (BD, MD, USA) was inserted gently into the lumen of the exposed trachea. The lungs were then lavaged with two separate 1-mL washes of sterile normal saline. The RBD-specific IgA titer of BAL samples was next evaluated by ELISA as described above with Goat anti-mouse IgA alpha chain-HRP (Abcam, Cambridge, UK, 1:3,000).

### Anti-RBD IgG measurements

RBD-specific antibody titers in serum samples collected from immunized animals with 50 μL of 1 × 10^6^ PFU/mL of vaccine were determined by indirect ELISA. Ninety-six-well microtiter plates were coated with 200 ng of purified RBD protein which was generated and expressed in 293F from the codon optimized RBD sequence of SARS-CoV-2 spike protein (GenBank accession number MN908947 and WEX63463.1) individually at 4°C overnight and blocked with 2% BSA for 2 h at 37°C. Diluted sera (1:100) were successively diluted in a 2-fold series and applied to each well for 1 h at 37°C, followed by incubation with goat antimouse, antihamster, or antihuman antibodies conjugated with HRP for 1 h at 37°C after three washes. The plate was developed using 3, 3′,5,5′-tetramethylbenzidine (TMB), followed by the addition of 2M H_2_SO_4_ to stop the reaction, and read at 450/630 nm by ELISA plate reader for final data acquisition.

### ELISPOT assay

ELISPOT assays were performed using mouse IFN-γ ELISPOT plates (DAKEWE, Shenzhen, China). Ninety-six-well ELISPOT plates precoated with capture antibody were blocked with RPMI-1640 for 10 min at room temperature. Briefly, a total of 10^6^ cells per well from lung tissues of C57BL/6 mouse immunized with 50 μL of 1 × 10^6^ PFU/mL of vaccine were plated into each well and stimulated for 20 h with pooled peptides of RBD of wild-type SARS-CoV-2 or variants (15-mer peptide with 11 amino acids overlap, cover the RBD_305–547_, Genscript). The spots were developed based on the manufacturer's instructions. PBS and cell stimulation cocktails from the kit were used as negative and positive controls, respectively. Spots were scanned and quantified by an ImmunoSpot CTL reader. Spot-forming units (SFUs) per million cells were calculated by subtracting the negative control wells.

### SARS-CoV-2 RNA quantification

Viral RNA levels in the lungs of challenged hamsters were detected by quantitative RT-PCR. Briefly, for quantification of viral levels and gene expression after challenge, RNA was extracted from homogenized organs or cultured cells using a QIAamp Viral RNA Mini Kit (Qiagen, Hilden, Germany) according to the manufacturer's instructions. Hamster tissue samples were homogenized by TissueLyser II (Qiagen, Hilden, Germany) in 1 mL of PBS. Subsequently, viral RNA quantification was conducted using a SARS-CoV-2 RT-PCR Kit (Wantai, Beijing, China) by measuring the copy numbers of the N gene.

### SARS-CoV-2 titration assay

Live virus titers in homogenized lung tissues and cell cultures were measured by the standard TCID_50_ method in Vero E6 cells seeded in 96-well plates. In brief, the samples were serially diluted, added to the 96-well plates and incubated with the Vero E6 cells for 1 h. Three days after incubation, the cytopathic effects were observed and used to calculate the viral titers.

### Histopathology

The lung tissues from challenged hamsters were fixed with 10% formalin for 48 h, embedded in paraffin and sectioned. Next, the fixed lung sections were subjected to hematoxylin and eosin (H&E) staining. Whole-slide images of the lung sections were captured by an EVOS M7000 Images System (Thermo Fisher). The standards for pathological score of lung tissues in this study are adapted and optimized from a recent study of SARS-CoV-2 infection in hamster model (25). In brief, H&E staining result of the whole lung tissue was analyzed for its severity of pathological change. The pathological score includes: (i) alveolar septum thickening and consolidation; (ii) hemorrhage, exudation, pulmonary edema, and mucous; (iii) recruitment and infiltration of inflammatory immune cells. For each issue, score related to the severity: 0 indicates no pathological change was observed, 1 indicates moderate pathological change, 2 indicates mild pathological change, 3 indicates severe pathological change, and 4 indicates very severe pathological change. In conclusion, scores of such three issues were added as the comprehensive lung pathological score of a lung tissue.

### Statistical analysis

Statistical significance was assigned when *P*-values were <0.05 using GraphPad Prism 8.0. The bars in this study represent the mean ± SD. The number of animals and independent experiments that were performed are indicated in the figure legends. Student's t-test (two groups) or one-way ANOVA (three or more groups) was used for comparison of normally distributed continuous variables. Two-way repeated-measures ANOVA was adopted for repeated data comparison. For multiple comparisons of three or more groups, Dunnett's multiple comparison test was used.

## Results

### Construction and immune response of the dNS1-XBB-RBD candidate

**Fig. 1.**
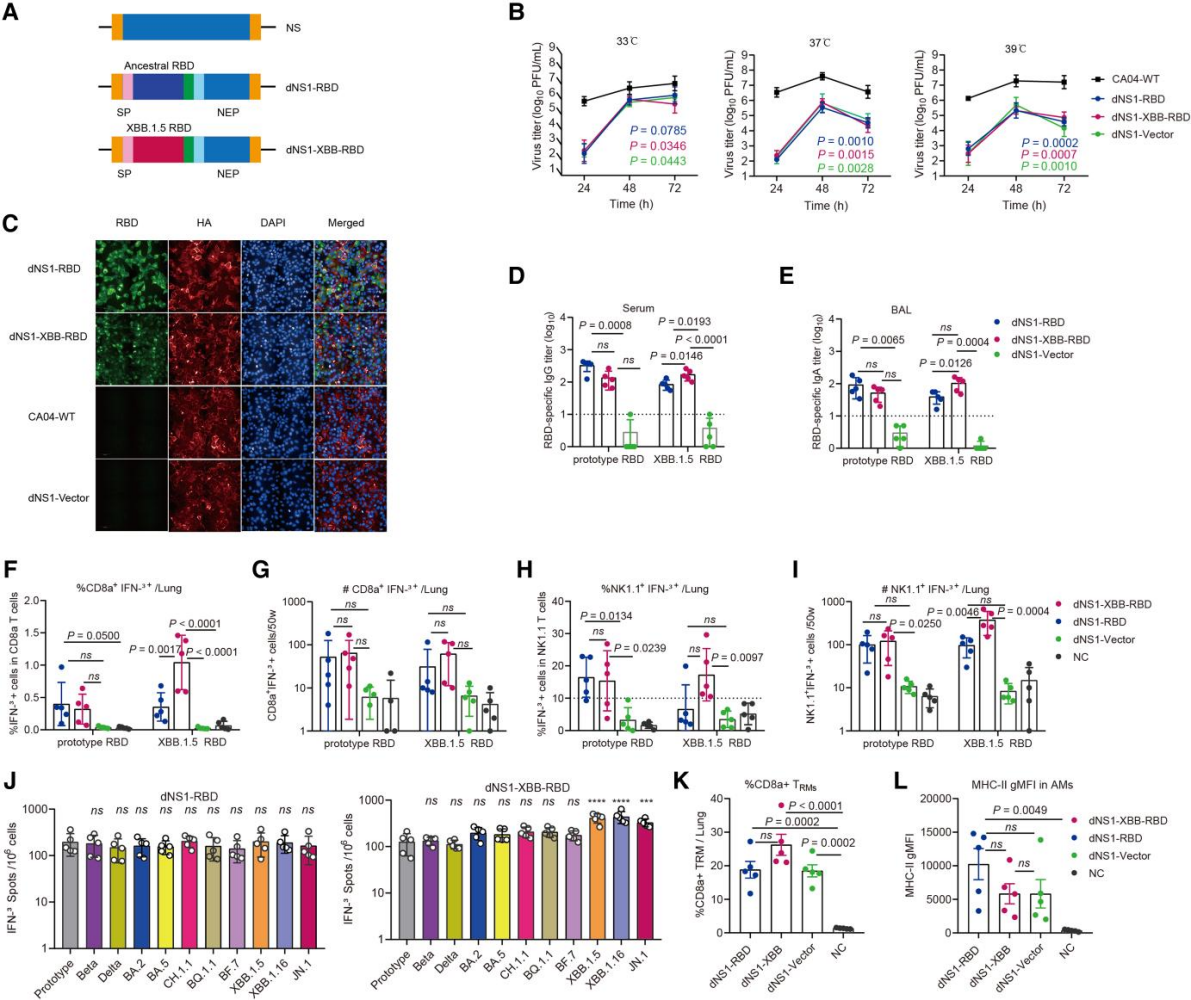
Construction and characterization of a recombinant live-attenuated influenza virus-based SARS-CoV-2 vaccine against XBB.1.5 variant. A) Construction of an mRNA-encoding plasmid that transcribes dNS1 with RBD-inserted mRNA. RBD, receptor-binding domain. The dNS1-RBD, constructed with ancestral RBD. The dNS1-XBB-RBD, constructed with XBB.1.5 RBD. B) Replication efficiency of the dNS1-RBD, dNS1-XBB-RBD, dNS1-Vector, and CA04-WT viruses varied with 33°C, 37°C, and 39°C conditions in MDCK cells. C) Confocal analysis of the RBD and HA protein expressed by the influenza vector in MDCK cells. Red fluorescence indicates the HA; green fluorescence indicates RBD. RBD-specific IgG levels (D) in serum and IgA levels (E) in BAL fluid were measured by ELISA for BALB/c mice vaccinated twice by dNS1-RBD, dNS1-XBB-RBD, or dNS1-Vector at weeks 2 and 4. Intranasal immunization with dNS1-RBD, dNS1-XBB-RBD, and dNS1-Vector activated T and NK cells at 7 days after vaccination. Statistical analysis plots for the percentage and cell numbers of CD8a + IFN-γ+ (F and G) and NK1.1 + IFN-γ+ (H and I) in the lung. J) IFN-γ ELISPOT assays for pulmonary lymphocytes from C57BL/6 mice at 2 weeks after two-dose vaccination with 2 weeks interval were assessed under prestimulation of various peptide pools covering the RBD of SARS-CoV-2 variants. Tissue-resident memory T cells in lungs and trained phenotype of alveolar macrophages were induced by dNS1-RBD, dNS1-XBB-RBD, and dNS1-Vector. Bar graph showing the frequency of CD8+ TRs (K) in lungs and the gMFI of MHC II (L) on AMs 14 days post a prime-boost regimen. *N* = 5 biologically independent mice per group. The dNS1-Vector, the dNS1-based influenza virus without RBD gene inserted. CA04-WT and dNS1-Vector was set as control here. Data are shown as mean ± SD. Two-way repeated-measures ANOVA with Dunnett’s multiple comparisons test (B) and ordinary one-way ANOVA multiple comparison (D, E, F, G, H, I, J, K, and L) were used for intergroup statistical comparisons. Asterisks indicate statistical significance (****P < 0.0001; ***P < 0.001; **P < 0.01; *P < 0.05; ns, not significant).

The dNS1-XBB-RBD candidate was constructed by inserting a gene encoding the receptor-binding domain (RBD) of the spike protein of the SARS-CoV-2 XBB.1.5 strain into the previously reported NS1-deleted backbone of H1N1 influenza virus California/4/2009 (CA04-dNS1) (Fig. 1A) as described (19). Notably, the dNS1-XBB-RBD vaccine exhibited a similar temperature-sensitive characteristic with dNS1-RBD, which significantly suppressed its replication and growth kinetics at 37°C and 39°C compared to 33°C in MDCK cells when compared to CA04-WT (Fig. 1B). This temperature sensitivity is desirable as it reduces the risk of influenza-associated adverse reactions in the lungs. Confocal analysis and Western blot confirmed the expression of the RBD and HA proteins in infected MDCK cells (Figs. 1C and S1). Nasal inoculation of New Zealand rabbits confirmed the attenuation of dNS1-XBB-RBD, as no weight loss or pathological injury was observed in nasal turbinate, trachea, or lung tissues at days 1, 8, 15, 16, and 29 postinoculation after triple-doses of 10^6^ PFU of dNS1-XBB-RBD or saline at 1-week interval (Fig. S2).

Additionally, the immune response to the dNS1-RBD and dNS1-XBB-RBD vaccines was compared by intranasally immunizing cohorts of mice with one or two doses. Similar to the previous study (19), samples of serum and bronchoalveolar lavage (BAL) were collected and measured 2 weeks following boosting. However, both the dNS1-RBD and dNS1-XBB-RBD vaccines failed to induce a strong systemic and mucosal antibody response against the prototype and XBB.1.5 strains, as no detectable neutralizing antibodies were produced (Figs. 1D and E). In intracellular IFN-γ expression analysis by flow cytometry, both dNS1-RBD and dNS1-XBB-RBD elicited a strong and rapid IFN-γ producing RBD-specific T-cell response in the lung, while the strength of the XBB.1.5 specific immune response for dNS1-RBD was weaker than that for dNS1-XBB-RBD at 7 days post single dose after stimulation using prototype and XBB.1.5 specific 15-mer RBD-peptide pools, respectively (Figs. 1F and G and S3). A similar phenomenon was found in NK cells (Fig. 1H and I), which indicated the rapid activation and proliferation for the RBD-specific T and NK cells in the murine lungs. Similarly, enzyme-linked immunosorbent spot (ELISpot) assays on isolated pulmonary lymphocytes stimulated with peptides containing key mutations found in major variants (including Beta, Delta, BA.2, BA.5, CH.1.1, BQ.1.1, BF.7, XBB.1.5, XBB.1.16, and JN.1) showed a similar RBD-specific T-cell response in the lungs for both vaccines (Fig. 1J). Interestingly, the dNS1-XBB-RBD vaccine induced higher levels of cell-mediated immunity against XBB.1.5, XBB.1.16, and JN.1 compared to dNS1-RBD, while no significant difference was observed in the antibody response. This highlights the importance of conserved and strong local T-cell responses in the respiratory tract for long-term and broad-spectrum protective effects.

Growing evidence supports the critical role of tissue-resident memory (TRM) T-cell responses in coordinating effective defense against reinfection in local tissues (Fig. S4) (26, 27). The memory effect of trained immunity is also thought to play a significant role in broad-spectrum anti-infection immunity (28). Alveolar macrophages (AMs) are key players in the innate pulmonary defense during respiratory infection, with high MHC II expression considered to be a trained phenotype (29). Vaccination with dNS1-RBD or dNS1-XBB-RBD 14 days after the second dose resulted in significantly elevated memory CD8 T cells with tissue-resident phenotype (CD69 + CD103+) and MHC II expression on AMs, similar to the CA04-dNS1 control group (Fig. 1K and L). These results support the idea that both dNS1-RBD and dNS1-XBB-RBD vaccines, when administered intranasally, can establish local and long-lasting broad-spectrum protection against SARS-CoV-2 variants tract by maintaining the local tissue-resident memory T-cell response and trained immunity in the respiratory tract.

### Broad protection against infection with the Beta and XBB.1.9.2.1 variants of SARS-CoV-2 in hamsters after intranasal immunization with dNS1-RBD or dNS1-XBB-RBD

**Fig. 2.**
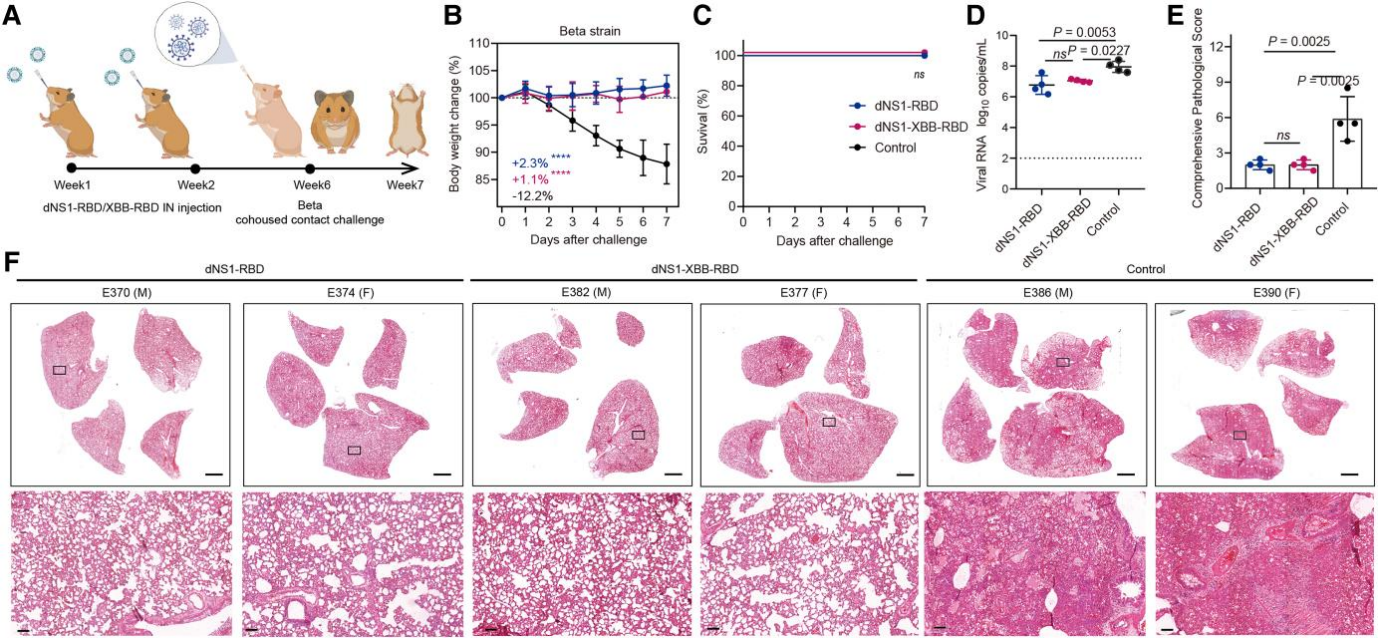
The dNS1-RBD and dNS1-XBB-RBD confer protection against SARS-CoV-2 Beta variant challenge in hamster. A) Timeline of vaccination and challenge experiments in Syrian hamsters. The hamsters were challenged by Beta variant of SARS-CoV-2 at 4 weeks after two-dose vaccination with cohoused transmission mode, with 8 animals in each groups. Weight changes (B), survival curves (C), lung viral RNA levels (D), and pulmonary pathological scores (E) of hamsters challenged by Beta variant were shown. The average weight loss of each group at 7 dpi is indicated as a colored number. The lung tissues were collected on day 5 after cohousing exposure for viral RNA and pathological analysis. Pulmonary pathological scores were determined based on the severity and percentage of injured areas for the whole lung tissue collected from the indicated animal. F) Representative H&E-stained lung sections from tested hamsters collected on day 5 after cohousing exposure. Views of the whole lung lobes (four independent sections) are presented in the above panel (scale bars, 2 mm), and the areas in the small black boxes are enlarged in the lower panel (scale bars, 100 μm). Dotted lines indicate the lower detection limit of the assay (D). Data are shown as mean ± SD. Two-way repeated-measures ANOVA with Dunnett’s multiple comparisons test (B), two-sided log-rank test (C), and ordinary one-way ANOVA multiple comparison (D and E) were used for intergroup statistical comparisons. Asterisks indicate statistical significance (****P < 0.0001; ***P < 0.001; **P < 0.01; *P < 0.05; ns, not significant).

**Fig. 3.**
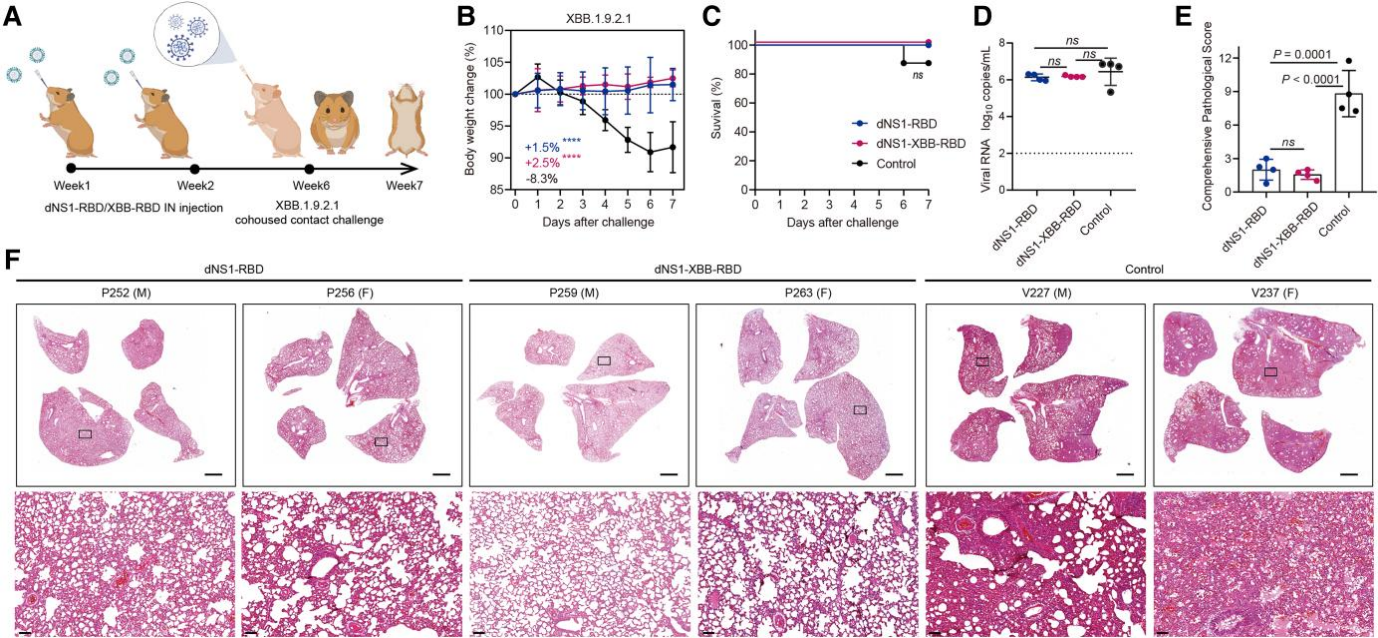
The dNS1-RBD and dNS1-XBB-RBD confer protection from weight loss and reduced pathology against SARS-CoV-2 XBB.1.9.2.1 variant challenge in hamster, not from virus replication. A) Timeline of vaccination and challenge experiments in Syrian hamsters. The hamsters were challenged by XBB.1.9.2.1 variant of SARS-CoV-2 at 4 weeks after two-dose vaccination with cohoused transmission mode, with eight animals in each groups. Weight changes (B), survival curves (C), lung viral RNA levels (D), and pulmonary pathological scores (E) of hamsters challenged by XBB.1.9.2.1 variant were shown. The average weight loss of each group at 7 dpi is indicated as a colored number. The lung tissue was collected on day 5 after cohousing exposure for viral RNA and pathological analysis. Pulmonary pathological scores were determined based on the severity and percentage of injured areas for the whole lung tissue collected from the indicated animal. F) Representative H&E-stained lung sections from tested hamsters collected on day 5 after cohousing exposure. Views of the whole lung lobes (four independent sections) are presented in the above panel (scale bars, 2 mm), and the areas in the small black boxes are enlarged in the lower panel (scale bars, 100 μm). Dotted lines indicate the lower detection limit of the assay (D). Data are shown as mean ± SD. Two-way repeated-measures ANOVA with Dunnett’s multiple comparisons test (B), two-sided log-rank test (C), and ordinary one-way ANOVA multiple comparison (D and E) were used for intergroup statistical comparisons. Asterisks indicate statistical significance (****P < 0.0001; ***P < 0.001; **P < 0.01; *P < 0.05; ns, not significant).

Syrian hamsters are a highly sensitive preclinical animal model commonly used to evaluate vaccine efficacy against both ancestral SARS-CoV-2 and emerging variants (25, 30, 31). The hamster model can mimic the predominant natural route of SARS-CoV-2 infection in humans and associated COVID-19-like lung damage and can support efficient viral transmission from inoculated hamsters to naïve hamsters by direct contact and via aerosols. In this study, we aimed to assess the ability of the dNS1-RBD and dNS1-XBB-RBD vaccines, based on ancestral and XBB.1.5 RBD, respectively, to provide protection against SARS-CoV-2 variants. Intranasal immunizations with dNS1-RBD and dNS1-XBB-RBD were conducted in a golden Syrian hamster interanimal transmission model, which simulates the primary route of SARS-CoV-2 infection, 4 weeks after the completion of the two-dose vaccination regimen (Figs. 2A and 3A). Virus-carrying hamsters (donors) were preinfected via inoculation of 1 × 10^4^ TCID_50_ of Beta variant or XBB.1.9.2.1 variant through the intranasal route. Each donor was then transferred to a new cage and cohoused with four vaccinated or control animals. One day after cohousing, donors were isolated from the cage, and the other hamsters were further observed.

For the Beta variant challenge, the control groups showed continuous body weight loss starting from 1 day postinfection (dpi), with a maximum weight loss of 12.2% at 7 dpi. However, all animals in the control groups survived after the Beta challenge. In contrast, the vaccinated groups did not exhibit substantial weight loss, with mean weight gains of +2.3% for the dNS1-RBD groups and +1.1% for the dNS1-XBB-RBD groups (Fig. 2B and C). The viral loads in the lung tissue of vaccinated hamsters were slightly lower than those in the control groups at 5 and 7 dpi, likely due to the absence of detectable neutralizing antibodies (Fig. S5). At the end of the experiment, lung damage at 5 and 7 dpi was quantitatively assessed using a comprehensive pathological scoring system and hematoxylin and eosin (H&E) staining examination. The control groups exhibited mild-to-moderate pulmonary consolidation, alveolar destruction, and diffuse inflammation. In contrast, both vaccinated hamsters were largely protected from lung damage caused by infection with the Beta variant. They showed minimal focal histopathological changes in the lung lobes, and the severity scores of lung pathology were significantly lower in the vaccinated hamsters compared to the controls (Figs. 2E and F and S6). This finding is consistent with previous experiments (19, 20) that demonstrated the independent protective effect of the dNS1-RBD vaccine against SARS-CoV-2 by restraining the inflammatory response, regardless of virus clearance. Thus, the newly developed dNS1-XBB-RBD vaccine also showed a nonantigen-specific protective effect against the Beta variant challenge.

We further assessed the protective effect of the dNS1-RBD and dNS1-XBB-RBD vaccine against XBB.1.9.2.1 variant in hamsters 4 weeks after immunization. The unvaccinated animals exhibited a maximum weight loss of 8.3% at 7 dpi, along with significant clinical symptoms induced by XBB.1.9.2.1 variant in hamsters, which differed from the clinical disease caused by Omicron strains in rodents (30, 32). In contrast, hamsters in both vaccinated groups exhibited an average weight increase of 1.5 to 2.5% compared to their baseline levels at the end of the 7-day follow-up (Fig. 3B). Furthermore, one out of eight unvaccinated cohoused animals succumbed to contact challenges with the XBB.1.9.2.1 variant (Fig. 3C). At 5 and 7 dpi, the viral RNA loads in the lung tissue of vaccinated hamsters remained slightly lower than those of the exposed control hamsters (Figs. 3D and S5). Histopathological examination revealed significantly higher pathological scores in the exposed control groups, indicating severe pulmonary disease with consolidated pathological lesions. Moreover, they showed intense interstitial pneumonia characterized by inflammatory cell infiltration in either a focally diffuse or multifocal distribution compared to both vaccine groups (Figs. 3E and F and S6). Additionally, there were no statistically significant differences in pulmonary pathology scorings among all vaccinated hamsters, although the dNS1-XBB-RBD groups showed relatively milder pathological changes when compared to dNS1-RBD vaccinated animals. These findings suggest that the XBB.1.5-specific immune response induced by dNS1-XBB-RBD, as observed earlier, contributes to these results and further demonstrate that this nasal dNS1-based vaccine can elicit a multidimensional protective immune mechanism, including, but not limited to, RBD-specific immune responses. In order to validate the nonantigen-specific protective effect, hamsters were challenge by Beta variants through cohoused exposure after inoculated by dNS1-RBD or dNS1-Vector (Fig. S7). The protective efficacy induced by the dNS1-Vector was comparable to that induced by dNS1-RBD at 7 dpi. Slightly enhanced protection was observed in hamsters vaccinated with dNS1-RBD, as reflected by the pulmonary pathology scorings. Consequently, intranasal immunization with the dNS1-XBB-RBD vaccine provided effective protection, comparable to the ancestral-targeted dNS1-RBD vaccines, against lung pathology induced by the antigenically distant XBB.1.9.2.1 variant in hamsters.

### Intranasal booster using dNS1-XBB-RBD vaccine confers mucosal and systemic immunity against SARS-CoV-2 variants

**Fig. 4.**
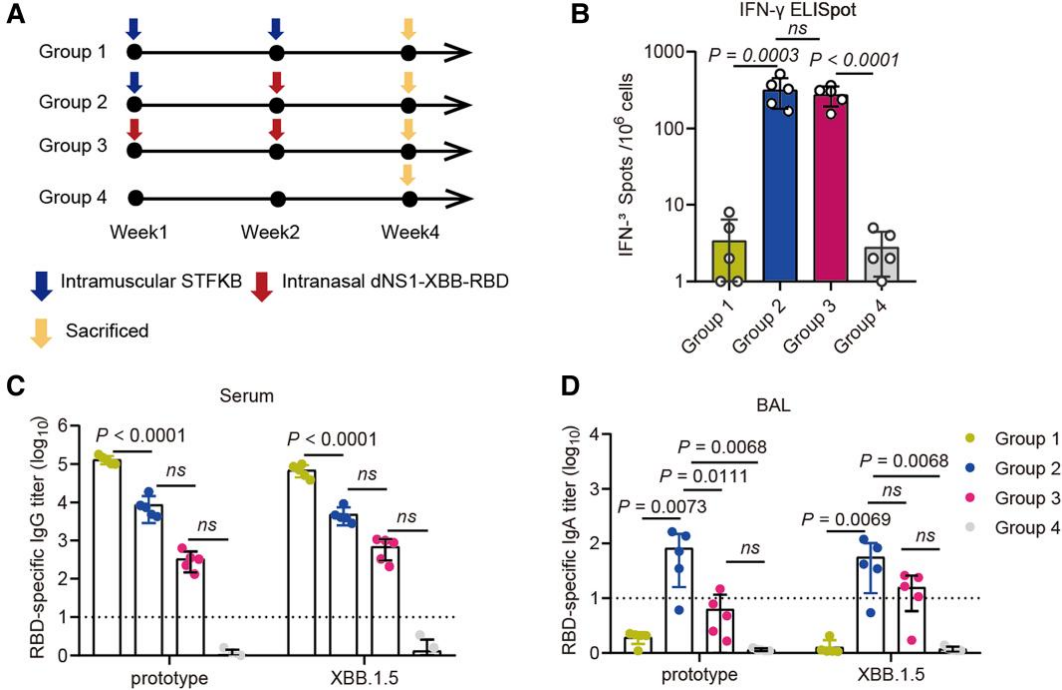
The immune response induced by dNS1-XBB-RBD as for booster immunization. A) Experimental design and schema, group 1: two doses of STFK, group 2: single dose of STFK and boost with dNS1-XBB-RBD, group 3: two doses of dNS1-XBB-RBD, group 4: control. B) IFN-γ ELISPOT assays for pulmonary lymphocytes from C57BL/6 mice were assessed under prestimulation of various peptide pools covering the RBD of XBB variant. RBD-specific IgG levels (C) in serum and IgA levels (D) in BAL fluid were measured by ELISA for BALB/c mice. Data are shown as mean ± SD. Ordinary one-way ANOVA multiple comparison (B–D) were used for intergroup statistical comparisons. Asterisks indicate statistical significance (ns, not significant).

Considering that the existing vaccines on the market are mainly intramuscular vaccines, identifying potential strategies to enhance local immunity following intramuscular (IM) immunization is essential. A sequential immunization strategy, incorporating STFKB—a SARS-CoV-2 subunit vaccine known for its potent induction of both humoral and cellular immune responses in peripheral blood—and dNS1-XBB-RBD boosting (33), was implemented to validate the enhancement of local immunity attributed to the boosting process (Fig. 4). Compared to mice that received STFKB alone (group 1), intranasal booster vaccination of dNS1-XBB-RBD at 14 days after the last IM vaccination (group 2), and only dNS1-XBB-RBD vaccination (group 3) led to a ∼90-fold increase in RBD-specific IFN-γ T-cell response in the lung (Fig. 4B). When mice in group 1 showed robust RBD-specific IgG response when compared to groups 2 and 3, mice with IN booster or only vaccination of dNS1-XBB-RBD vaccine could both induce significantly higher level of IgA in BAL against the prototype and XBB.1.5 strains. And mice in group 2 seems to show increase in IgA antibody response as compared with mice that received dNS1-XBB-RBD vaccine alone, although not statistically significant (Fig. 4C and D). These data suggest that by combining IM immunization with an intranasal dNS1-XBB-RBD boost, high levels of local lung immune responses were achieved.

## Discussion

Due to the rapid mutation and transient immunity of the population, it appears that COVID-19 epidemics in the future will follow a year round recurring mini-wave pattern rather than seasonal surges observed with influenza viruses (34). As intramuscular vaccines have been widely administered worldwide, boosting with intranasal vaccines can provide more comprehensive immune protection in the respiratory location (35). Moreover, local cellular immunity and trained immunity in the respiratory tract are considered to be relatively broad-spectrum, which is beneficial for addressing challenges posed by SARS-CoV-2 variants (36, 37). In a previous publication, we described a nasally delivered live-attenuated influenza-vectored vaccine, dNS1-RBD, which targets the RBD of the SARS-CoV-2 prototype and has been approved for emergency use in China on 2022 December 2, under the name *Pneucolin* (19). This vaccine candidate exhibited promising efficacy and a favorable safety profile, particularly in elderly individuals or those with underlying chronic diseases. It also conferred broad-spectrum protection against symptomatic COVID-19 caused by the SARS-CoV-2 Omicron variant in adults of all ages regardless of underlying medical conditions. This protection was observed with both primary vaccination and heterologous booster doses in a Phase 3 clinical trial (ChiCTR2100051391). The dNS1-RBD vaccine provided protection against SARS-CoV-2 infection without inducing significant levels of neutralizing antibodies, while due to the local T-cell and trained immunity response. The protective mechanism of this intranasal vaccine differs from that of traditional vaccines and challenges our previous understanding of vaccine immunity.

In response to the emergence of XBB variants and sublineages, we developed an updated vaccine called dNS1-XBB-RBD expressing the XBB subvariant. In this study, we assessed the immune response of dNS1-RBD and dNS1-XBB-RBD vaccines at the systemic, mucosal, and trained immunity levels. We also evaluated their protective activity against the Beta and XBB.1.9.2.1 variants in Syrian hamster model. The nasal spray formulations of dNS1-based vaccines induced moderate systemic IgG and mucosal IgA responses in mice. Additionally, these vaccines elicited robust tissue-resident memory T cells and trained immunity, providing immune protection across the respiratory tract. Notably, the dNS1-XBB-RBD vaccine generated a slightly higher adaptive immune response against the matched antigen than the dNS1-RBD vaccine. These findings are consistent with previous studies demonstrating that many T-cell peptide epitopes are conserved among SARS-CoV-2 strains. Furthermore, spike-specific T cells induced by COVID-19 vaccination have been shown to recognize the spike peptides of antigenically distinct SARS-CoV-2 variants, including XBB.1.5 and other Omicron variants (38–40). Updating vaccines may be especially important for systemically delivered vaccines that induce a poor mucosal and T-cell response in the local respiratory tract. This warrants a quantitative correlation between substantial neutralizing antibodies against emerging variants and protective efficacy. However, the antibody-independent protection mechanisms related to the local T-cell response and trained immunity would not be influenced by this updating design. In addition, the dNS1-vector virus control can truly induce similar innate and trained immune response, and provide inferior protection against SARS-CoV-2 compared to dNS1-RBD vaccine. It seems that the non-RBD-specific immune response induced by the dNS1-vector is an important component of vaccines for broad-spectrum protective immunity.

Furthermore, an XBB.1.9.2.1 challenge model based on hamsters was developed for the first time, demonstrating that the intranasal administration of the updated dNS1-XBB-RBD vaccine can provide protection against Beta and XBB variant challenge. At the same time, it was proven that the intranasal administration of the dNS1-RBD vaccine constructed with the ancestral RBD confers broad-spectrum protection against the emerging XBB.1.9.2.1 strain. As expected, the viral loads in lung tissue of Beta and XBB.1.9.2.1 challenged hamsters were not observed for either vaccinated groups but may be due to a prominent restraining inflammatory response locally. In the lower respiratory tract, no significant differences were observed in protection against the matched and unmatched dNS1-based vaccines.

In the previous studies, the immune response to the LAIV, FluMist^@^, was found to be multifaceted and does not necessarily involve a serum antibody response (41, 42). A human challenge trial of LAIVs also suggested that a low antibody response was not directly associated with low protective efficacy (43). In general, the induction of mucosal antibodies and a local T-cell response by LAIVs was similar to those induced by dNS1-RBD in adults (44, 45). Therefore, additional immune correlate studies in humans are clearly needed especially for the nonsystemic respiratory viruses such as influenza viruses or SARS-CoV-2, within a narrow window of time before adaptive immune responses are fully marshaled (46).

There are two main limitations in this study. First, while our study provides valuable insights into the broadly protective efficacy of both dNS1-RBD and dNS1-XBB-RBD, it is important to note that the duration of protection needs to be confirmed in more animal models in the future. Second, we did not evaluate the potential impact of natural or acquired immunity on the nature of T-cell immunity induced by this vaccine against SARS-CoV-2. In conclusion, the above results indicate that the dNS1-RBD and dNS1-XBB-RBD vaccine could be effective against XBB epidemic variants, suggesting that a nasal spray SARS-CoV-2 vaccine based on NS1-deleted influenza virus vectors should be a promising broad-spectrum vaccine strategy against COVID-19 in future. Furthermore, our proposed flu-based vaccine can also offer protection against seasonal human influenza viruses, representing a potential approach to fight against a possible “twindemic” of COVID-19 and flu in the future.

## Supplementary Material

pgae183_Supplementary_Data

## Data Availability

All data are included in the manuscript and/or supporting information.
