# Supplementary material for: Intranasal influenza-vectored COVID-19 vaccines confer broad protection against SARS-CoV-2 XBB variants in hamsters
Source: PNAS Nexus. 2024 May 3;3(5):pgae183. doi: 10.1093/pnasnexus/pgae183 (PMC11118774; doi:10.1093/pnasnexus/pgae183)
Supplement: pgae183_Supplementary_Data [file pgae183_supplementary_data.docx]

**Intranasal influenza-vectored COVID-19 vaccines confer broad protection against SARS-CoV-2 XBB variants in hamsters**

**Authors:** Junyu Chen ^1^^, 2^^, 4^, Congjie Chen ^1, 4^, Lunzhi Yuan ^1, 2,^ ^4^, Yaode Chen ^1, 4^,Xijing Wang^1, 4^, Ningxin Tang ^1^, Dongmei Wei ^1^, Xiangzhong Ye ^3,^ *, Yixin Chen^1, 2,^ *, Ningshao Xia ^1, 2,^ *

**Affiliations:**

^1^ State Key Laboratory of Vaccines for Infectious Diseases, Xiang An Biomedicine Laboratory, Department of Laboratory Medicine, School of Public Health, School of Life Sciences, Xiamen University, Xiamen 361102, China

^2^ National Institute of Diagnostics and Vaccine Development in Infectious Diseases, State Key Laboratory of Molecular Vaccinology and Molecular Diagnostics, Collaborative Innovation Center of Biologic Products, National Innovation Platform for Industry-Education Intergration in Vaccine Research, Xiamen University, Xiamen 361102, China

^3^ Beijing Wantai Biological Pharmacy Enterprise Co., Ltd., Beijing, 102206, China

^4^ These authors contributed equally.

* Corresponding authors: yexiangzhong@ystwt.com (X.Y.);yxchen2008@xmu.edu.cn (Y.C.); nsxia@xmu.edu.cn (N.X.).

**This PDF file includes:**

Supplementary Fig. 1-7


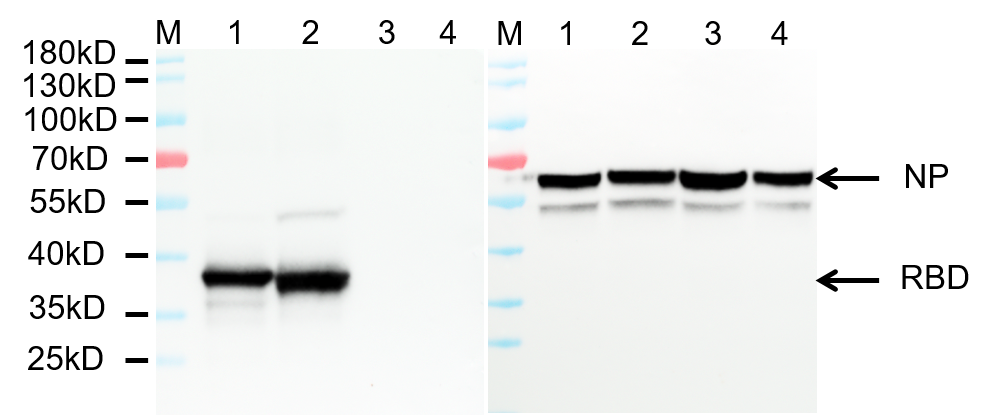


**Supplementary Figure 1.** Immunoblot analysis of RBD and NP expression in denatured cell lysate samples 48 h after infection by dNS1-RBD (1), dNS1-XBB-RBD (2) , CA04-WT (3) and dNS1-Vector (4).


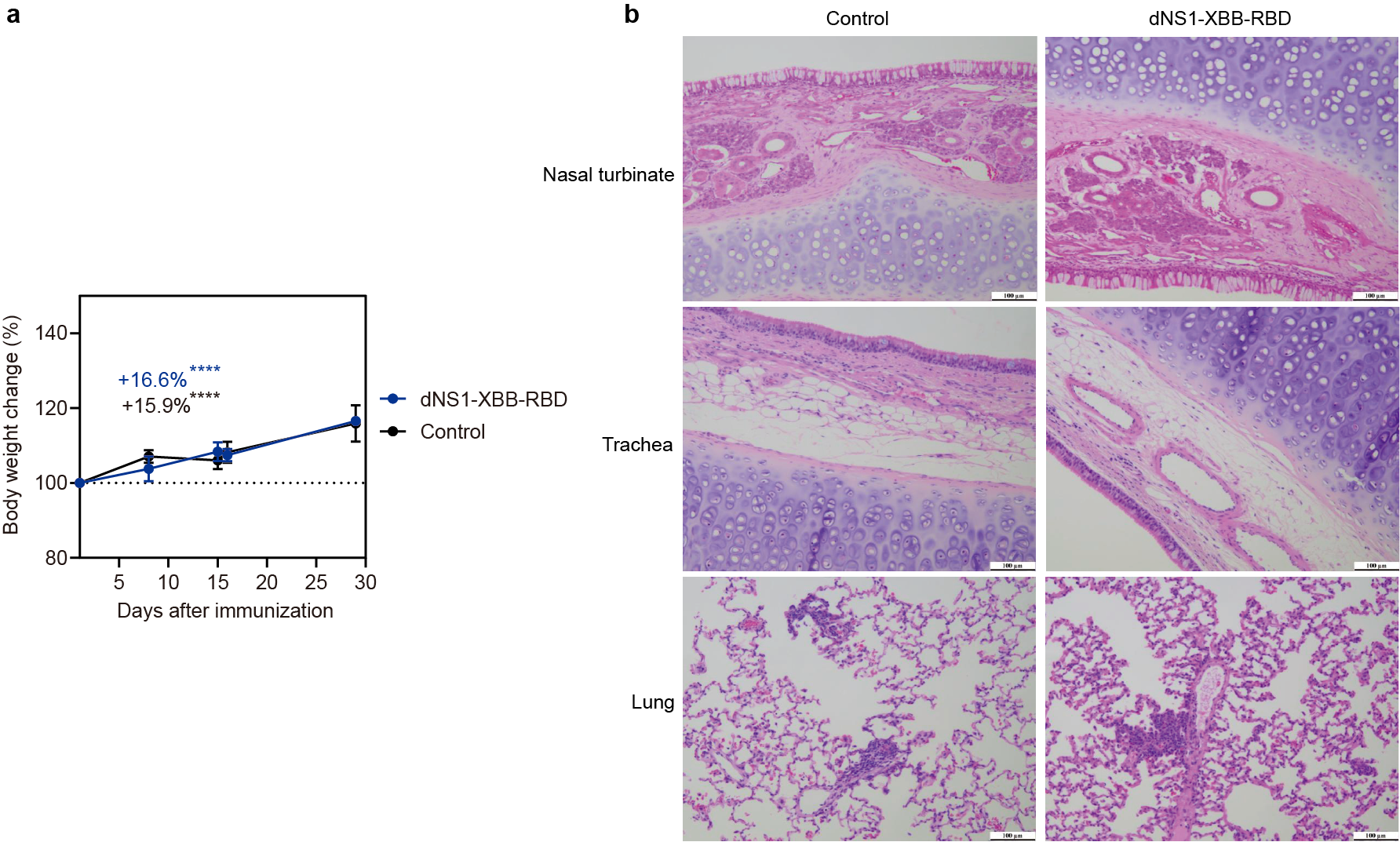


**Supplementary Figure 2.** Pathogenicity evaluation of dNS1-XBB-RBD in New Zealand rabbits. Two groups (n=6) of New Zealand rabbits were immunized with a triple-dose 1×10^6^ PFU of the dNS1-XBB-RBD vaccines or saline through the intranasal route at one–week interval. (*a*) Weight changes were recorded at 1, 8, 15, 16, 29 dpi. (*b*) Histopathological evaluations of the nasal turbinate, trachea and lungs from the two groups at day 16 post administration. Data are shown as mean ±SD. Two-way repeated-measures analysis of variance (ANOVA) with Dunnett`s multiple comparisons test were used for intergroup statistical comparisons. Asterisks indicate statistical significance (****p < 0.0001; ***p < 0.001; **p < 0.01; *p < 0.05; ns, not significant).


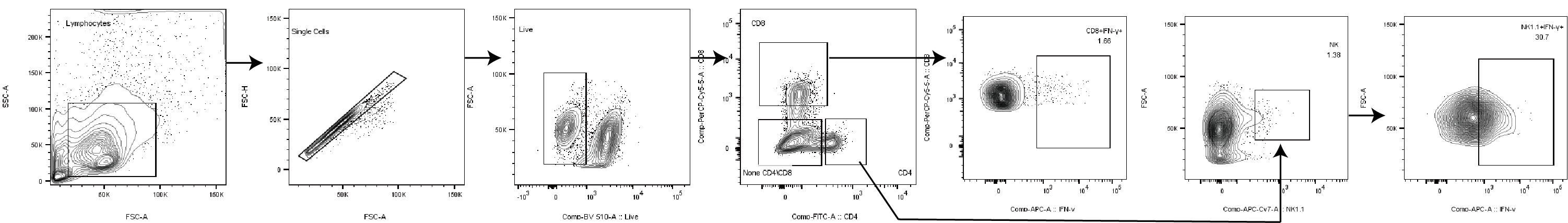


**Supplementary Figure 3.** Flow cytometry gating strategies, Related to Fig 1. Gating strategy for NK cells and IFN-γ+ CD8+ T cells in the lung, remove doublets and CD45+ cell gating are not shown.


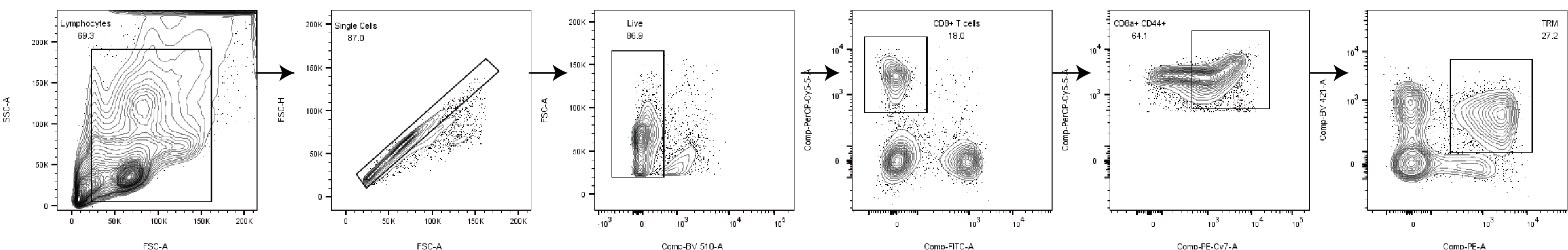


**Supplementary Figure 4.** Flow cytometry gating strategies, Related to Fig 1. Gating strategy for CD44+ CD8a+ T cells and TRM in the lung, remove doublets is not shown.


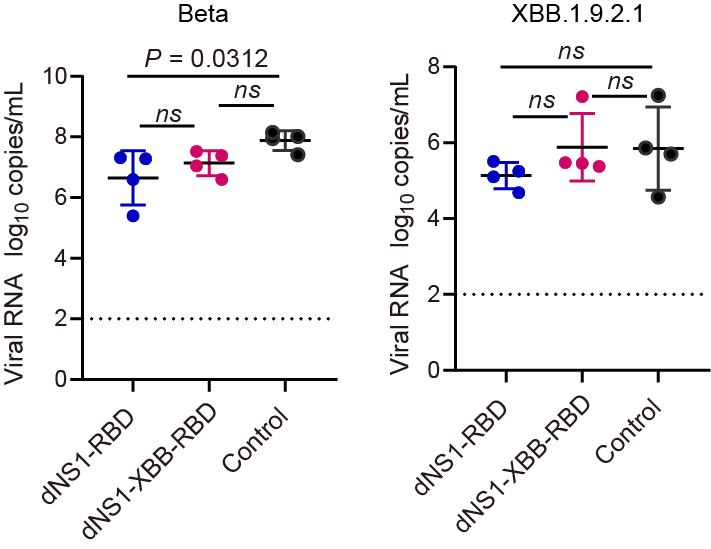


**Supplementary Figure 5.** Lung viral RNA levels of hamsters challenged by Beta and XBB.1.9.2.1 variant at 4 weeks after two dose vaccination with co-housed transmission mode were shown. The lung tissue were collected on day 7 after cohousing exposure for viral RNA and pathological analysis. Data are shown as mean ±SD. Ordinary one-way ANOVA multiple comparison were used for intergroup statistical comparisons. Asterisks indicate statistical significance (****p < 0.0001; ***p < 0.001; **p < 0.01; *p < 0.05; ns, not significant).


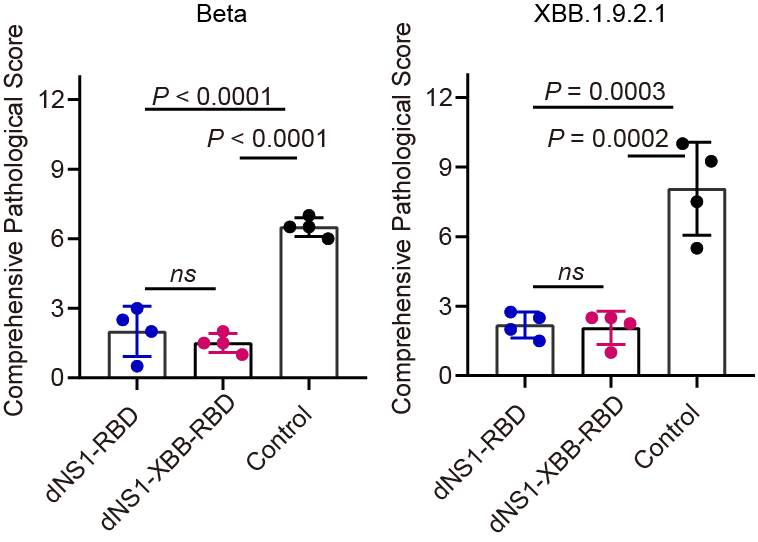


**Supplementary Figure 6.** Pulmonary pathological scores of hamsters challenged by Beta and XBB.1.9.2.1 variant at 4 weeks after two dose vaccination with co-housed transmission mode were shown. The lung tissue were collected on day 7 after cohousing exposure for viral RNA and pathological analysis. Ordinary one-way ANOVA multiple comparison were used for intergroup statistical comparisons. Asterisks indicate statistical significance (****p < 0.0001; ***p < 0.001; **p < 0.01; *p < 0.05; ns, not significant).


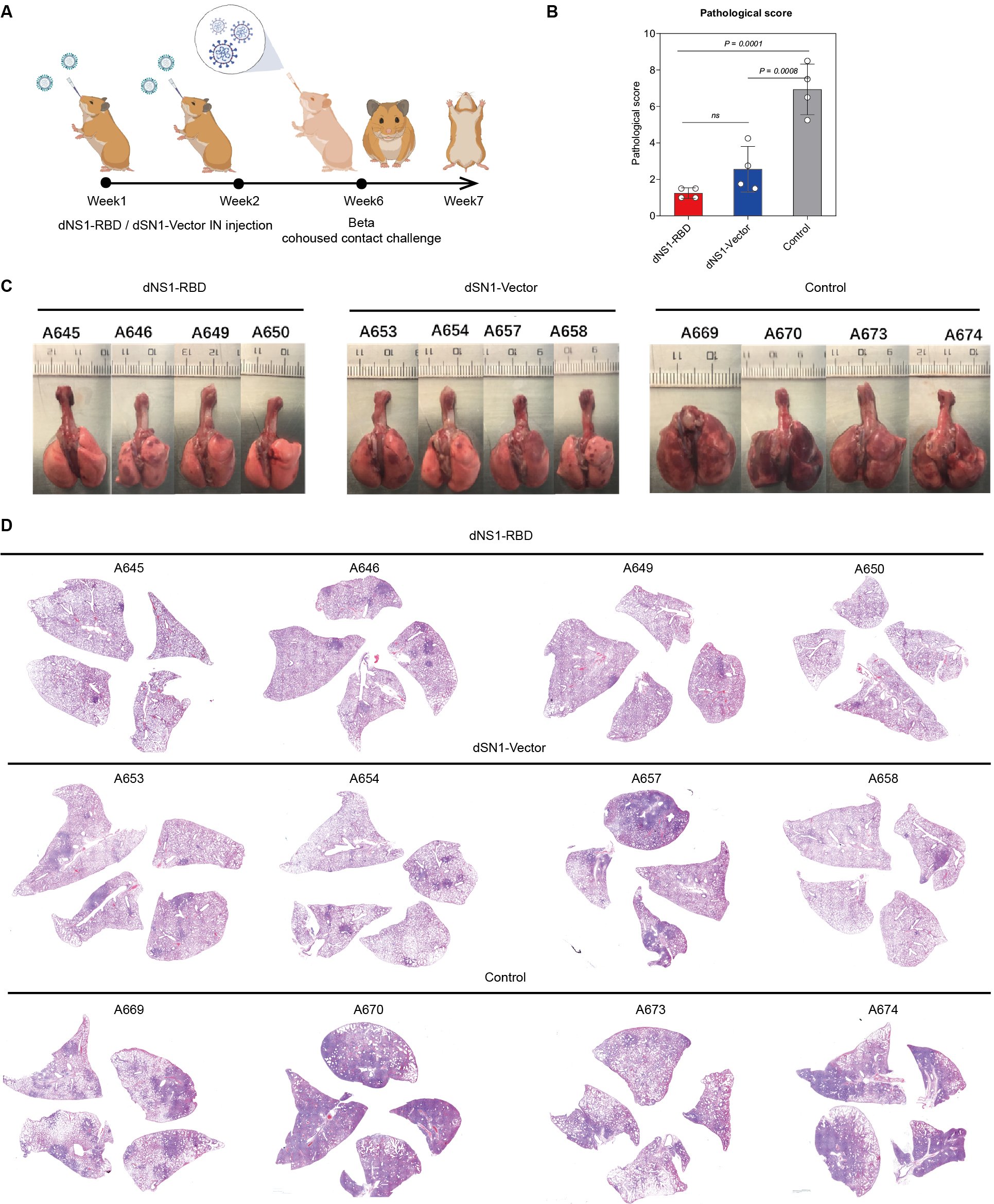


**Fig.7** The dNS1-RBD and dNS1-Vector confer protection against SARS-CoV-2 Beta variant challenged in hamster. (A) Timeline of vaccination and challenge experiments in Syrian hamsters. The hamsters were challenged by Beta variant of SARS-CoV-2 at 4 weeks after two dose vaccination with co-housed transmission mode, with 4 animals in each groups. (B) Pulmonary pathological scores of hamsters challenged by Beta variant were shown. Pulmonary pathological scores were determined based on the severity and percentage of injured areas for the whole lung tissue collected from the indicated animal. (C) Gross observations of lung tissues from challenged hamsters. (D) Representative H&E-stained lung sections from tested hamsters collected on day 7 after cohousing exposure. Data are shown as mean ±SD. Ordinary one-way ANOVA multiple comparison (B) were used for intergroup statistical comparisons. Asterisks indicate statistical significance (****p < 0.0001; ***p < 0.001; **p < 0.01; *p < 0.05; ns, not significant).
